# Genetic basis of hypercholesterolemia in adults

**DOI:** 10.1038/s41525-021-00190-z

**Published:** 2021-04-14

**Authors:** Seyedmohammad Saadatagah, Merin Jose, Ozan Dikilitas, Lubna Alhalabi, Alexandra A. Miller, Xiao Fan, Janet E. Olson, David C. Kochan, Maya Safarova, Iftikhar J. Kullo

**Affiliations:** 1 Department of Cardiovascular Medicine, Mayo Clinic, Rochester, MN USA; 2 Department of Health Sciences Research, Mayo Clinic, Rochester, MN USA; 3 Gonda Vascular Center, Mayo Clinic, Rochester, MN USA

**Keywords:** Risk factors, Genetic testing, Medical genomics, Dyslipidaemias

## Abstract

We investigated monogenic and polygenic causes of hypercholesterolemia in a population-based cohort, excluding secondary hypercholesterolemia, and using an established framework to identify pathogenic variants. We studied 1682 individuals (50.2 ± 8.6 years, 41.3% males) from southeast Minnesota with primary hypercholesterolemia (low-density lipoprotein cholesterol (LDL-C) ≥155 mg/dl in the absence of identifiable secondary causes). Familial hypercholesterolemia (FH) phenotype was defined as a Dutch Lipid Clinic Network (DLCN) score ≥6. Participants underwent sequencing of *LDLR, APOB*, and *PCSK9*, and genotyping of 12 LDL-C-associated single-nucleotide variants to construct a polygenic score (PGS) for LDL-C. The presence of a pathogenic/likely pathogenic variant was considered monogenic etiology and a PGS ≥90th percentile was considered polygenic etiology. The mean LDL-C level was 187.3 ± 32.3 mg/dl and phenotypic FH was present in 8.4% of the cohort. An identifiable genetic etiology was present in 17.1% individuals (monogenic in 1.5% and polygenic in 15.6%). Phenotypic and genetic FH showed poor overlap. Only 26% of those who met the clinical criteria of FH had an identifiable genetic etiology and of those with an identifiable genetic etiology only 12.9% met clinical criteria for FH. Genetic factors explained 7.4% of the variance in LDL-C. In conclusion, in adults with primary hypercholesterolemia, 17.1% had an identifiable genetic etiology and the overlap between phenotypic and genetic FH was modest.

## Introduction

Hypercholesterolemia is a major risk factor for atherosclerotic cardiovascular disease (ASCVD)^1,2^. It is estimated that 95 million U.S. adults age 20 or older have an elevated cholesterol level with only half on lipid-lowering treatment^3^. Both genetic and lifestyle factors are known to predispose to hypercholesterolemia^4,5^. However, prior studies attempting to delineate the genetic basis of hypercholesterolemia included individuals already diagnosed (clinically) with familial hypercholesterolemia (FH) or those referred to lipid clinics. The reported prevalence of monogenic and polygenic etiology in such cohorts ranged from 1.7%–50% and 20%–30%, respectively^6–15^. These estimates are affected by the referral bias inherent in such cohorts, inclusion of individuals with secondary forms of hypercholesterolemia, and variable application of guidelines to ascertain pathogenic variants in FH genes.

To address these limitations, we assessed the genetic basis of hypercholesterolemia in a cohort of individuals from the community who did not have a secondary cause for hypercholesterolemia. Pathogenic/likely pathogenic (P/LP) variants were identified by a molecular geneticist in a Central Laboratory Improvement Amendment (CLIA)-certified laboratory. We defined “phenotypic” FH, based on the Dutch Lipid Clinic Network (DLCN) criteria^16^ and “genetic” FH was defined as presence of a P/LP variant or elevated polygenic score (PGS) for low-density lipoprotein cholesterol (LDL-C). Additionally, we assessed the overlap between phenotypic and genetic forms of FH.

## Results

### Participant characteristics and phenotypic FH

**Fig. 1:**
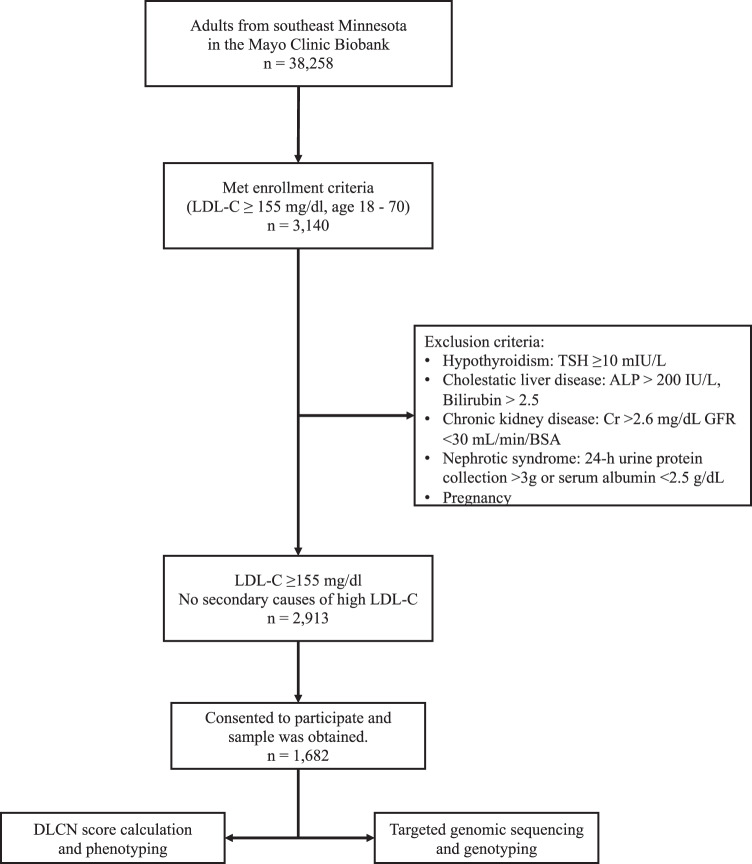
Selection of the study cohort. ALP alkaline phosphatase, BSA body surface area, Cr creatinine, DLCN Dutch lipid clinic network, GFR glomerular filtration rate, LDL-C low-density lipoprotein cholesterol, TSH thyroid stimulating hormone.

Out of 38,258 Mayo Biobank participants, 2913 met study criteria and 1682 consented and participated in the study (Fig. 1). The mean (±SD) age was 50.2 ± 8.6 years, 41.3% were males, 97.2% were non-Hispanic whites. The mean LDL-C level was 187.3 ± 32.3 mg/dl, 590 (35.1%) participants had severe hypercholesterolemia (LDL-C ≥190 mg/dl), 818 (48.6%) had LDL-C ≥95th age-sex-specific percentile, and 372 (22.1%) had LDL-C ≥99th age-sex-specific percentile. Phenotypic FH, defined as DLCN ≥6, was present in 142 (8.4%) (Table 1). Physical stigmata of FH were noted in only three patients, including two with tendon xanthomata and one with arcus cornealis.

**Table 1:** Participant characteristics (*n* = 1682).

Demographics	
Age years, mean ± SD	50.2 ± 8.6
Male, *n* (%)	695 (41.3%)
Whites, *n* (%)	1635 (97.2%)
Cardiovascular risk factors	
LDL-C mg/dl, mean ± SD	187.3 ± 32.3
LDL-C ≥190 mg/dl, *n* (%)	590 (35.1%)
LDL-C ≥95th percentile, *n* (%)	818 (48.6%)
LDL-C ≥99th percentile, *n* (%)	372 (22.1%)
Triglyceride mg/dl, mean ± SD	154. 9 ± 67.7
HDL-C mg/dl, mean ± SD	54.8 ± 14.6
BMI kg/m^2^, mean ± SD	29.55 ± 7.72
Diabetes, *n* (%)	171 (10.2%)
Hypertension, *n* (%)	604 (35.9%)
LDL-C on LLT, *n* (%)	65 (3.9%)
Premature ASCVD	
Personal history, *n* (%)	307 (18.3%)
Family history, *n* (%)	287 (17.1%)
Phenotypic FH	
Unlikely, *n* (%)	926 (55.0%)
Possible, *n* (%)	614 (36.6%)
Probable/definite, *n* (%)	142 (8.4%)
Genetic etiology	
Polygenic, *n* (%)	262 (15.6%)
Monogenic FH, *n* (%)	25 (1.5%)

*ASCVD* atherosclerotic cardiovascular disease, *BMI* body mass index, *FH* familial hypercholesterolemia, *HDL-C* high-density lipoprotein cholesterol, *LDL-C* low-density lipoprotein cholesterol, *LLT* lipid-lowering therapy.

### Monogenic and polygenic etiologies

**Fig. 2:**
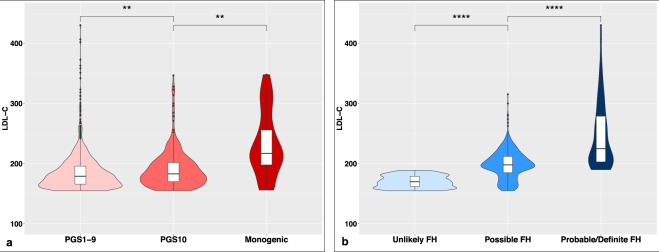
Distribution of LDL-C levels in the study population. **a** Distribution of LDL-C levels based on genotype, and **b** based on phenotype. The boxplots are embedded in the density plots. The central line represents median, box limits represent upper and lower quartiles, the vertical lines represent 1.5× quartile range, and points represent outliers. In the left plot, PGS1–9 indicates those with the PGS in the 1st to 9th decile. PGS10 represents those with the PGS in the top decile (polygenic etiology of hypercholesterolemia) and Monogenic represents those with a P/LP variant in *LDLR, APOB* or *PCSK9*. On the right side, DLCN criteria are used for categorizing cases as Unlikely FH: DLCN <3, Possible FH: 3 ≤ DLCN <6, and Probable/Definite FH: DLCN ≥6. FH familial hypercholesterolemia, LDL-C low-density lipoprotein cholesterol, PGS polygenic score (number refers to PGS decile). *t*-test is used for comparison. *P-value < 0.05, **P-value < 0.01, ***P-value < 0.001, ****P-value < 0.0001 and ns = non-significant.

Sequencing of *LDLR, APOB* and *PCSK9* identified 1300 variants of which 200 were rare and functional variants. Based on American College of Medical Genetics (ACMG) criteria, 25 participants (1.5%) had P/LP variants. The number (percentage) of participants with variants in *LDLR, APOB, and PCSK9* were 18 (72%), 6 (24%), and 1 (4%) respectively (Supplementary Table 2). The mean PGS in the study cohort was 0.97 ± 0.21 (Supplementary Fig. 2). Among participants without monogenic FH, 262 (15.6%) had polygenic hypercholesterolemia (PGS ≥1.16, which corresponds to 90th percentile of PGS distribution in the UK-Whitehall II cohort). The mean LDL-C level in carriers of P/LP variants (monogenic FH) was 232.04 ± 54.78 mg/dl, significantly higher than those with PGS >90th percentile (polygenic FH, 191.35 ± 32.18 mg/dl; *P*-value < 0.01), which in turn was higher than in those without these two conditions (185.72 ± 31.20 mg/dl; *P*-value < 0.01). (Fig. 2) There were no significant differences between triglyceride and high-density lipoprotein cholesterol levels between these three categories (Supplementary Fig. 3). Characteristics of clinical and genetic subgroups are provided in Supplementary Tables 3 and 4. Only 1.1% of the variance in LDL-C was explained by clinical and demographic factors, whereas 7% was explained by genetic factors (Supplementary Tables 6, 7).

### Overlap between phenotypic and genetic FH

**Fig. 3:**
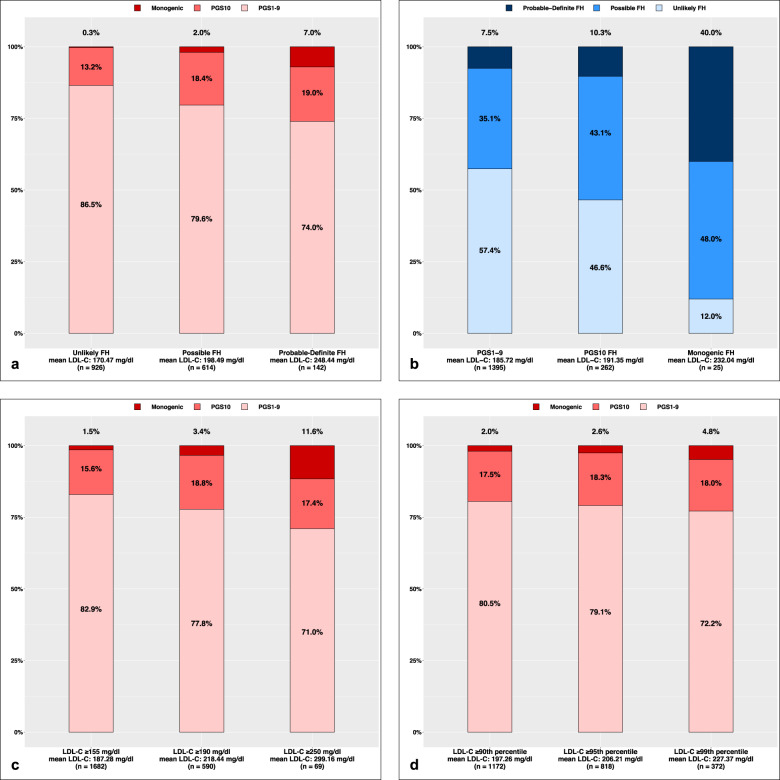
Overlap of monogenic/polygenic FH and FH ascertained by clinical criteria. **a** Proportion of individuals in FH phenotypic categories who had an identifiable genetic etiology. **b** Proportion of individuals with polygenic or monogenic etiology for hypercholesterolemia who met the clinical criteria for FH. **c** Proportion of individuals in different LDL level who had an identifiable genetic etiology. **d** Proportion of individuals in different LDL percentiles who had an identifiable genetic etiology. FH familial hypercholesterolemia, LDL-C low-density lipoprotein cholesterol, PGS polygenic score. PGS1–9 indicates those with the PGS in the 1st to 9th decile. PGS10 represents those with the PGS in the top decile (polygenic etiology of hypercholesterolemia) and Monogenic represents those a P/LP variant in *LDLR, APOB* or *PCSK9*. DLCN criteria are used for the categorization of cases as Unlikely FH: DLCN <3, Possible FH: 3 ≤ DLCN <6, and Probable/Definite FH: DLCN ≥6.

**Fig. 4:**
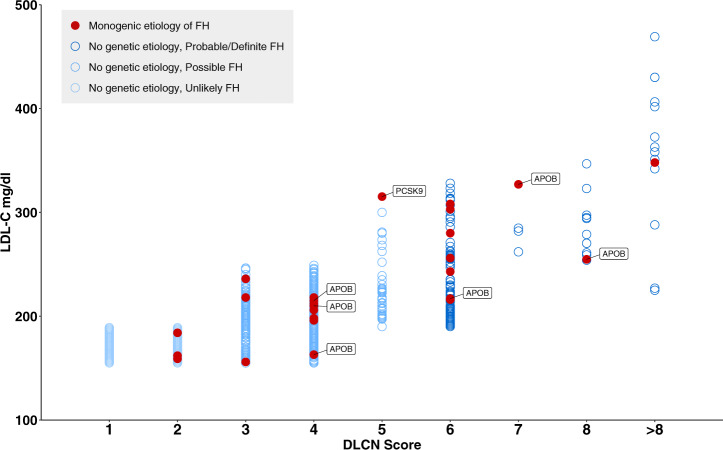
Distribution of monogenic etiology across categories of DLCN score and corresponding LDL-C levels. Cases with P/LP *APOB* or *PCSK9* variants are labeled and the remaining had P/LP *LDLR* variants. DLCN Dutch lipid clinic network, FH familial hypercholesterolemia, LDL-C low-density lipoprotein cholesterol.

The overlap between those with a genetic etiology and those with phenotypic FH is summarized in Fig. 3. Among those with phenotypic FH (DLCN ≥6, *n* = 142), 26.0% had a genetic etiology—7.0% monogenic and 19.0% polygenic. The distribution of monogenic FH cases across the spectrum of DLCN scores is depicted in Fig. 4. Among those with a genetic etiology (*n* = 287), 12.9% had phenotypic FH; including 40.0% (10 out 25) with a monogenic etiology and 10.3% (27 out of 262) with a polygenic etiology, respectively. When considering only the LDL-C level, a monogenic etiology was present in 3.4% and 11.6% of those with LDL-C ≥190 mg/dl (*n* = 590) and LDL-C ≥ 250 mg/dl (*n* = 69), respectively. When participants were categorized based on their age-sex-specific LDL-C percentile, monogenic etiology of FH was present in 2.6% and 4.8% of those with LDL-C ≥95th (*n* = 818) and ≥99th (*n* = 372) percentile, respectively.

## Discussion

The main findings of our study of adults with primary hypercholesterolemia (i.e., LDL-C ≥155 mg/dl without an identifiable secondary cause) were: (1) a genetic etiology was present in 17.1%—monogenic in 1.5% and polygenic in 15.6%; (2) 8.4% met DLCN criteria for FH; (3) there was poor overlap between the presence of a genetic etiology and clinically diagnosed FH. Our results suggest that genetic testing could be helpful for ascertaining FH cases and highlight the heterogeneity in the definitions of FH.

The frequency of monogenic FH was 3.4% in individuals with LDL-C ≥190 mg/dl, nearly double that (1.7%) reported by Khera et al.^17^ and higher than 2.5% reported by Abul-Husn et al.^7^. Several factors could account for differences in prevalence such as inclusion of patients with secondary hypercholesterolemia in the previous studies and differences in how P/LP variants were identified. When considering age-and sex-specific percentiles, monogenic etiology of FH was present in 2.6% and 4.8% of participants with LDL-C ≥95th and ≥99th percentile, respectively. The prevalence increased to 7.0% in individuals with phenotypic FH (DLCN score ≥6). Our findings suggest that the yield of genetic testing in those with LDL-C ≥190 mg/dl is higher if secondary causes are ruled out and also if additional DLCN criteria are present. These data can inform clinicians regarding the expected yield of genetic testing in individuals with severe hypercholesterolemia.

Conversely, more than half (60.0%) of those with a monogenic etiology did not meet the DLCN criteria for FH. In the study by Abul-Husn et al.^7^ 76.3% of individuals with a P/LP variant in FH genes did not have phenotypic FH. These observations indicate that clinical criteria are not sensitive in identifying individuals with monogenic FH and highlight the potential value of population genetic screening for FH case detection. Since presence of a P/LP variant is associated with increased coronary heart disease risk even after adjustment for LDL-C^17^, such knowledge will be useful in individualized care of affected individuals and also facilitate cascade testing of family members^18^.

The overall prevalence of a polygenic etiology (i.e., PGS ≥90th percentile of the general population) in our study was 15.6%, 19.0% in those with clinical FH, comparable with the prevalence of 20–30% in another cohort with clinical FH^19^. A polygenic etiology was thus 10-fold more common than monogenic etiology in individuals with phenotypic FH. Increasing the number of single nucleotide variants (SNVs) in a PGS for LDL-C may categorize additional individuals as having a polygenic etiology. Our results suggest that a PGS for LDL-C may be useful in the clinical setting to establish the etiology of hypercholesterolemia including phenotypic FH. Conversely, only 10.3% of those with polygenic etiology had a DLCN ≥6.

In our study cohort of adults with primary hypercholesterolemia, variance in LDL-C explained by genetic factors (7.4%) was greater than that explained by clinical/demographic variables (1.1%). The latter is not unexpected since LDL-C is a heritable trait and heritable factors account for up to 60% of its inter-individual variance^20^. In a substantial proportion, a genetic etiology was not identified, highlighting the need to identify additional etiologic factors for primary hypercholesterolemia. Such factors may include additional as yet uncharacterized monogenic/polygenic determinants, gene-gene and gene-environment interactions, and epigenetic effects^21^. In particular, in individuals with phenotypic FH, no P/LP variant in *LDLR, APOB* and *PCSK9* and a low polygenic score for LDL-C, a novel monogenic etiology may be present^22^. In such probands, family studies and further gene discovery may be warranted. In the present study, among those with phenotypic FH, only 13 (9.1%) individuals had low PGS ( <20th percentile).

Our findings could have implications for clinical management of patients with FH. Given the poor overlap between genetic and phenotypic FH, genetic testing could be helpful for complete ascertainment of FH patients. Since more than half of the individuals with P/LP variants did not meet clinical criteria for FH, increased use of genetic testing as well as population scale genomic initiatives may be needed to identify individuals with monogenic FH who would otherwise go undetected. A substantial proportion of patients with clinical FH has polygenic hypercholesterolemia. Further research is needed to assess the utility of cascade testing in patients with clinical FH who do not have monogenic etiology^23^ and to identify new genetic etiologies in those with clinical FH, no P/LP variant and a low PGS.

Pathogenic variants were identified by a molecular geneticist in a CLIA-certified laboratory, based on ACMG guidelines instead of solely relying on computational tools or databases. Phenotypic FH was ascertained using a validated algorithm for FH followed by manual review. The majority (96.1%) of patients were treatment naïve at the time the highest LDL-C was recorded and in the remainder, we imputed pre-treatment LDL-C based on the statin type and dose^24^. A limitation is lack of ethnic diversity in the study cohort. Participants were residents of Southeast Minnesota who participated in the Mayo Clinic Biobank and there may be a healthy participant and survivor bias. The *APOB* c.10580G > A variant was present at high frequency in our study cohort despite absence of relatedness among carriers, suggesting a potential founder effect. The variant is enriched in populations of Amish descent^25^ and was also reported to be at a similar frequency in two other large cohorts^7,26^. At the time of design of this study, the available PGS for LDL-C included 12 LDL-C-associated SNVs; however, additional variants associated with LDL-C have been identified since^27^. A PGS incorporating a greater number of LDL-C-associated variants would have explained a greater amount of variance in LDL-C and identified a greater proportion of our cohort as having a polygenic etiology. Nonetheless, we were able to infer that a polygenic etiology is present in at least 15.6% of cases with primary hypercholesterolemia and is much more frequent that monogenic etiology.

In conclusion, in adults with primary hypercholesterolemia (i.e., LDL-C ≥ 155 mg/dl), a genetic etiology was present in 17.1% (monogenic in 1.5% and polygenic in 15.6%) and genetic factors explained 7% of inter-individual variance in LDL-C. Given the modest overlap between the presence of a genetic etiology and phenotypic FH, genetic testing could be helpful in identifying FH cases who might otherwise remain undetected.

## Methods

### Participant recruitment

This study was approved by the Mayo Clinic Institutional Review Board. We identified Mayo Clinic Biobank^28^ participants from southeast Minnesota who were alive, aged between 18 and 70 years, and had hypercholesterolemia in the absence of a secondary cause. Hypercholesterolemia was defined as LDL-C level ≥155 mg/dl (~4 mmol/l) based on DLCN criteria^16^; secondary causes included hypothyroidism, cholestatic liver disease, severe kidney disease, nephrotic syndrome, and pregnancy detected within a 1-year window around the index date (first date of LDL-C ≥155 mg/dl). Case selection and exclusion criteria have been previously reported^29^ are also depicted in a flow chart (Fig. 1). Of 38,258 Mayo Biobank participants from southeast Minnesota, 1682 participants met inclusion criteria and consented to the study and were included in the final analysis.

### Targeted sequencing

DNA of participant was sent to the Baylor College of Medicine Human Genome Sequencing Center, a CLIA-certified facility, for sequencing exons of the three FH genes (*LDLR*, *APOB*, *PCSK9*) and genotyping of 12 single-nucleotide variants (SNVs) associated with LDL-C (Supplementary Table 1). Details of sequencing methods and quality control metrics of the sequencing data have been previously described^29,30^. All three genes had >99% of targeted bases sequenced to redundant coverage of ≥20. Sequence data were analyzed by the Mercury 3.4 pipeline^31^ (additional details of variant calling are described in Supplementary Method). The data from Illumina HiSeq were converted from bcl file to FASTQ file by Illumina bcl2fastq 1.8.3 software, and mapped to the hg19 human genome reference by the Burrows-Wheeler Aligner^32^. Both SNVs and copy number variants (CNV) were called using Atlas-SNP and Atlas-indel, respectively (additional details of CNV detection are provided in Supplementary Method). Variants passing quality control were mapped to gene loci using SeattleSeqAnnotation138. Principal component analysis of genetic ancestry and qualitative comparison to self-reported ancestry were performed as a part of quality control analyses (details provided in the Supplementary Method and Supplementary Fig. 1). The self-reported ancestry and genetically determined ancestry matched in our study participants.

### Monogenic etiology: identifying P/LP Variants

The final variant annotation was based on ACMG/Association of Medical Pathology (ACMG/AMP) criteria^30,33^. Variants in *LDLR, APOB* and *PCSK9* meeting the following criteria were identified using InterVar^34^: (a) functional variants— missense, stop-gain, etc. (b) frequency of <0.1% in ExAC^35^ or gnomAD^36^, and (c) satisfying criteria for pathogenicity listed in the ACMG guideline^33^. Additional review included: (a) databases, i.e., ClinVar^37^, Leiden Open Variation Database^38^ and Human Gene Mutation Database^39^, (b) relevant literature, and (c) clinical features reported in the electronic health record (EHR).

### Polygenic etiology: polygenic score for LDL-C

A previously validated 12-SNV PGS^19^ was used to measure the polygenic component of elevated LDL-C level (Supplementary Table 1). For each individual, a PGS for LDL-C-was calculated using a weighted sum of the effect alleles at the 12 SNVs. The weights used were the corresponding per-effect allele beta coefficients reported in a genome wide association study meta-analysis^19,29^. The PGS calculated for each individual was compared to the distribution of the score in the Whitehall II cohort^19^ and a PGS ≥90th percentile (i.e., PGS ≥1.16) was considered as polygenic hypercholesterolemia, as in the derivation cohort, the mean LDL-C level of those with PGS ≥90th percentile was 190 mg/dl, which is the threshold for defining severe hypercholesterolemia^19^.

### Phenotypic FH

The previously validated SEARCH algorithm^40^ was used to extract DLCN scores from the EHR. Participants were classified as “definite”, “probable”, “possible” and “unlikely” FH based on a DLCN score >8, 6–8, 3–5, and <3, respectively. Phenotypic FH was defined as DLCN score ≥6. The EHRs of individuals with DLCN score ≥6 and those with monogenic FH were manually reviewed to confirm the presence of clinical criteria for FH. For individuals reporting statin use at the index date, untreated LDL-C level was imputed based on the type and dose of the statin, ascertained from the EHR, using a dynamic coefficient^24^. Premature ASCVD was considered as coronary heart disease, cerebrovascular disease, or peripheral artery disease in males before age 55 years and females before age 65 years^40^. In addition to using actual LDL-C level, we also estimated sex-, age-specific LDL-C percentile based on recent data derived from a white population^41^.

### Statistical analysis

Categorical variables are presented as counts (percentages), and continuous variables as mean ± SD. Group comparison for categorical and numerical variables was done using chi-square, *t*-test, and analysis of variances test, as appropriate. The amount of inter-individual variance in LDL-C explained by genetic factors and clinical and demographic factors (age, sex, race, body mass index, diabetes and family history of hypercholesterolemia) was estimated using a multivariable linear regression analysis. The details of regression models are provided in the supplementary material. Statistical analyses were performed using RStudio version 1.2.5033 (RStudio, Inc., Boston, MA). All tests were two-sided, and *P*-values < 0.05 were considered statistically significant.

### Reporting summary

Further information on experimental design is available in the Nature Research Reporting Summary linked to this paper.

## Supplementary information

Supplementary Information

Reporting Summary

## Data Availability

The dataset generated and/or analyzed during the current study are available from the corresponding author on reasonable request. All eMERGE III data are available on dbGaP using “phs001616.v2.p2” accession code. More details could be found using the following link https://www.ncbi.nlm.nih.gov/projects/gap/cgi-bin/study.cgi?study_id=phs001616.v2.p2.
