# Author Correction: Genetic basis of hypercholesterolemia in adults

**DOI:** 10.1038/s41525-021-00222-8

**Published:** 2021-06-29

**Authors:** Seyedmohammad Saadatagah, Merin Jose, Ozan Dikilitas, Lubna Alhalabi, Alexandra A. Miller, Xiao Fan, Janet E. Olson, David C. Kochan, Maya Safarova, Iftikhar J. Kullo

**Affiliations:** 1 Department of Cardiovascular Medicine, Mayo Clinic, Rochester, MN USA; 2 Department of Health Sciences Research, Mayo Clinic, Rochester, MN USA; 3 Gonda Vascular Center, Mayo Clinic, Rochester, MN USA

**Keywords:** Risk factors, Genetic testing, Medical genomics, Dyslipidaemias

Correction to: *npj Genomic Medicine* 10.1038/s41525-021-00190-z, published online 14 April 2021

The original version of this Article contained an error in the spelling of the author Lubna Alhalabi, which was incorrectly given as Lubna Alhabi.

This has now been corrected in both the PDF and HTML versions of the Article.

