# Supplementary material for: Genetic basis of hypercholesterolemia in adults
Source: NPJ Genom Med. 2021 Apr 14;6:28. doi: 10.1038/s41525-021-00190-z (PMC8046820; doi:10.1038/s41525-021-00190-z)
Supplement: Supplementary file 1 — Supplementary Information [file 41525_2021_190_MOESM1_ESM.pdf]

SUPPLEMENTAL MATERIAL

Genetic Basis of Hypercholesterolemia in Adults

Seyedmohammad Saadatagah, Merin Jose, Ozan Dikilitas, Lubna Alhabi, Alexandra A. Miller, Xiao Fan, Janet E. Olson, David C. Kochan, Maya Safarova, Iftikhar J. Kullo.

- **Supplementary Note 1**. Pipeline to analyze sequencing data and call variants in *LDLR*, *APOB* and *PCSK9*.
- **Supplementary Note 2**. Detection of Copy Number Variants.
- **Supplementary Note 3**. Principal Component Analysis for Ancestry.
- **Supplementary Figure 1**. PCA Plots for our study population.
- **Supplementary Figure 2**. Distribution of PGS in the study population.
- **Supplementary Figure 3**. Distribution of triglyceride and HDL-C in the study population.
- **Supplementary Table 1**. Variants used in polygenic score.
- **Supplementary Table 2.** A summary of LP/P variants identified in the study.
- **Supplementary Table 3**. Participant characteristics based on categories of phenotypic FH.
- **Supplementary Table 4**. Participant characteristics based on genetic etiology.
- **Supplementary Table 5**. Comparison of mean LDL-C level in different participant subgroups
- **Supplementary Table 6**. Association of clinical and demographic factors with LDL-C level.
- **Supplementary Table 7**. LDL-C level in different genetic categories.

Supplementary Note 1.
Pipeline to analyze sequencing data and call variants in *LDLR*, *APOB* and *PCSK9*. We downloaded the recalibrated binary sequencing alignment map (BAM) files (*n*=2,347) from DNANexus using the 'ds' command line client. For cohort analysis, GATK v3.5 best practice (https://software.broadinstitute.org/gatk/guide/) was used for calling SNVs and Indels simultaneously. For each sample, we used 'HaplotypeCaller' in the GATK package to generate an intermediate genomic gVCF file (in gVCF mode) with a standard min confidence threshold of 10 for variant calling. The interval list of the captured regions (3,651 regions with a total length of 537,864 bp) was specified in this step. Then, using 'GenotypeGVCFs', we performed the multi-sample join aggregation step and merged the records together at each position of the input gVCFs. We then filtered SNVs and Indels separately using the 'hard filters' based on the GATK guidelines, including 'QD<2.0, FS>60.0, MQ<40.0, MQRankSum <-12.5, and ReadPosRankSum<-8.0' for SNVs, and 'QD<2.0, FS>200.0, and ReadPosRankSum<-20.0' for Indels. We used 'VCFTools' (https://vcftools.github.io/index.html) to process the VCF file generated by GATK. We excluded non-variants and sites that have a filter tag from the generated VCF file (including SNVs and Indels), extracted variants in autosomal and X chromosomes, and removed sites with 3+ alleles. The number of variants according to the genotype call rate indicated that there is no significant change of number of variants at different genotype call rates.

Supplementary Note 2.
Detection of Copy Number Variants. CNV calls were made via Atlas-CNV, an in-house software that combines outputs from XHMM and the GATK Depth of coverage tool. (PMID: 20644199, PMID: 24763994) Atlas-CNV infers the presence of CNVs from normalized coverage differences to other

samples in the same sequencing batch and refines these predictions with a pair of quality control metrics. (PMID: 30890783) CNV calls, if present, were confirmed by orthogonal technology: Multiplex Ligation-dependent Probe Amplification (MRC-Holland). Detected CNVs were then manually reviewed and required to have a minimum of two contiguous exons for reporting. (PMID: 31447099).

Supplementary Note 3.

Principal Component Analysis for Ancestry. Using the R package SNPRelate, principal component analysis (PCA) was performed by the eMERGE coordinating center on the multisample variant call file (VCF) of the entire eMERGEseq cohort. (PMID: 31447099) There were a total of 65,050 variants. We excluded SNVs with MAF < 5%, or a missing rate > 5% and pruned at an LD threshold (r) of 0.84. This resulted in the selection of 1,571 SNPs. PCA plot for our study population (n = 1,682) depicted in Supplementary Figure 1.

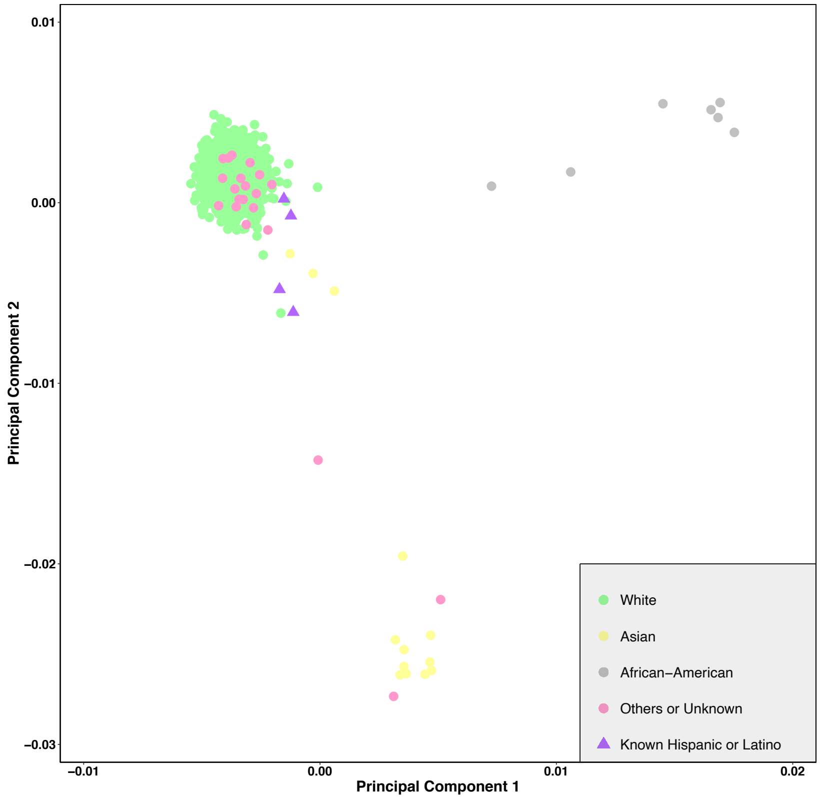

Supplementary Figure 1. PCA Plot for our study population (n=1,682)

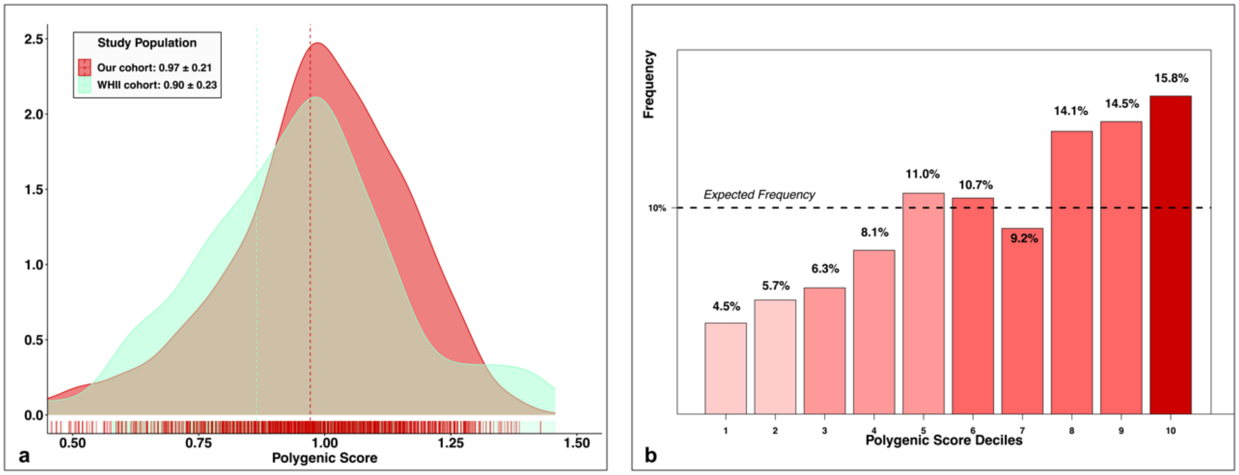

Supplementary Figure 2. Distribution of PGS for LDL-C in the study population. **a**) Comparison of distribution of PGS in our cohort with WHII population. **b)** Frequency of PGS deciles in our study in comparison to expected frequencies in the general population.

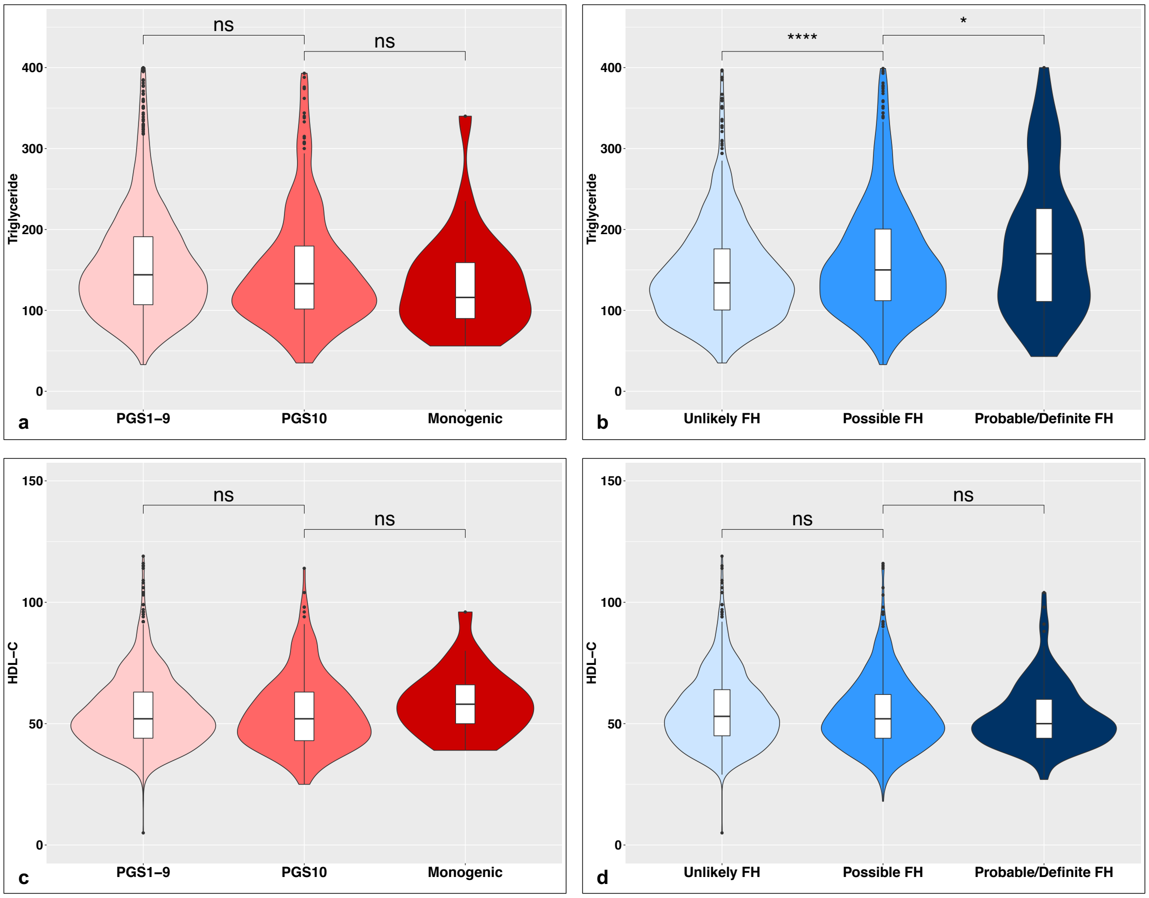

Supplementary Figure 3. Distribution of triglyceride and HDL-C in the study population. Distribution of triglyceride (a and b) and HDL-C (c and d) level based on the genotype (a and c) and phenotype (b and d). The boxplots are embedded in the density plots. The central line represents median, box limits represent upper and lower quartiles, the vertical lines represent 1.5 × quartile range, and points represent outliers. In the left plot, PGS1-9 indicates those with the PGS in the 1st to 9th decile. PGS10 represents those with the PGS in the top decile (polygenic etiology of hypercholesterolemia) and Monogenic represents those with a P/LP variant in *LDLR, APOB* or *PCSK9*. On the right side, DLCN criteria are used for categorizing cases as Unlikely FH: DLCN < 3, Possible FH: 3 ≤ DLCN < 6, and Probable/Definite FH: DLCN ≥ 6. Abbreviations, HDL-C, high-density lipoprotein cholesterol. * *P*-value < 0.05, ** *P*-value < 0.01, *** *P*-value < 0.001, **** *P*-value < 0.0001 and ns means non-significant.

Supplementary Table 1. Variants used in calculating a polygenic score for LDL-C.

**Variant ID Position**	**Genotype**
rs2479409	chr1 g.55504650G>A
rs629301	chr1 g.109818306G>T
rs1367117	chr2 g.21263900G>A
rs4299376	chr2 g.44072576G>T
rs3757354	chr6 g.16127407C>T
rs1800562	chr6 g.26093141G>A
rs1564348	chr6 g.160578860T>C
rs11220462	chr11 g.126243952G>A
rs8017377	chr14 g.24883887G>A
rs6511720	chr19 g.11202306G>T
rs429358	chr19 g.45411941T>C
rs7412	chr19 g.45412079C>T

Supplementary Table 2. A summary of LP/P variants identified in the study.

Series	Variant	Alteration	Classification	LDL-C $_{mg/dl}$
1	APOB, c.10580G>A	(p.Arg3527Gln)	P	163
2	APOB, c.10580G>A	(p.Arg3527Gln)	P	210
3	APOB, c.10580G>A	(p.Arg3527Gln)	P	215
4	APOB, c.10580G>A	(p.Arg3527Gln)	P	217
5	APOB, c.10580G>A	(p.Arg3527Gln)	P	255†
6	APOB, c.10580G>A	(p.Arg3527Gln)	P	327
7	LDLR, c.1238C>T	(p.Thr413Met)	LP	212
8	LDLR, c.131G>A	(p.Trp44*)	P	218
9	LDLR, c.1432G>A	(p.Gly478Arg)	LP	256
10	LDLR, c.1444G>A	(p.Asp482Asn)	LP	216
11	LDLR, c.1444G>A	(p.Asp482Asn)	LP	308
12	LDLR, c.1474G>A	(p.Asp492Asn)	LP	243
13	LDLR, c.1586+5G>A	‡	LP	162
14	LDLR, c.1640T>C	(p.Leu547Pro)	LP	348
15	LDLR, c.1860G>A	(p.Trp620*)	P	303
16	LDLR, c.2029T>C	(p.Cys677Arg)	LP	236
17	LDLR, c.420G>C	(p.Glu140Asp)	LP	280
18	LDLR, c.542C>G	(p.Pro181Arg)	LP	159
19	LDLR, c.782G>T	(p.Cys261Phe)	LP	206
20	LDLR, c.796G>A	(p.Asp266Asn)	LP	184
21	LDLR, c.796G>A	(p.Asp266Asn)	LP	156†
22	LDLR, c.796G>A	(p.Asp266Asn)	LP	196
23	LDLR, c.798T>A	(p.Asp266Glu)	LP	218
24	LDLR, c.862G>A	(p.Glu288Lys)	LP	198
25	PCSK9, c.644G>A	(p.Arg215His)	LP	315†

* stop codon mutation. † Imputed LDL-C level after considering statin type and dose. ‡ Splice site variant.

Supplementary Table 3. Participant characteristics based on categories of phenotypic FH (n = 1682).

	**Unlikely FH (n = 926)**	**Possible FH (n = 614)**	**Probable/ Definite FH (n = 142)**	***P*-value**
Age, year	50.45 ± 8.68	50.08 ± 8.20	49.66 ± 9.33	0.489
Male, n (%)	400 (43.2%)	249 (40.6%)	46 (32.4%)	0.046
Non-Whites, n (%)	21 (2.3%)	24 (3.9%)	2 (1.4%)	0.093
BMI ≤ 25 kg/m^2, n (%)	188 (20.3%)	94 (15.3%)	23 (16.2%)	0.021
BMI 25-30 kg/m^2, n (%)	355 (38.3%)	227 (37.0%)	46 (32.4%)	
BMI >30 kg/m^2, n (%)	383 (41.4%)	293 (47.7%)	73 (51.4%)	
Diabetic, n (%)	67 (7.2%)	73 (11.9%)	31 (21.8%)	<0.001
Hypertensive, n (%)	310 (33.5%)	228 (37.1%)	66 (46.5%)	0.008
Premature atherosclerotic event, n (%)	7 (0.8%)	203 (33.1%)	97 (68.3%)	<0.001
Never smoked, n (%)	577 (63.1%)	339 (55.9%)	84 (59.6%)	0.080
Quit smoking, n (%)	286 (31.3%)	228 (37.7%)	46 (32.6%)	
Current smoking, n (%)	51 (5.6%)	39 (6.4%)	11 (7.8%)	
LDL-C mg/dl	170.47±9.90	198.49±23.0	248.4±57.85	<0.001
LDL-C <190 mg/dl, n (%)	926 (100%)	166 (27.0%)	0 (0.0%)	<0.001
LDL-C 190-249 mg/dl, n (%)	0 (0.0%)	440 (71.7%)	81 (57.0%)	
LDL-C ≥250 mg/dl, n (%)	0 (0.0%)	8 (1.3%)	61 (43.0%)	
Triglyceride mg/dl	144.9±60.23	164.2±70.78	180.1±85.52	<0.001
HDL-C mg/dl	55.5±14.84	54.0±14.49	53.3±13.23	0.070
Polygenic score ≥90th percentile, n (%)	122 (13.2%)	113 (18.4%)	27 (19.0%)	<0.001
Monogenic FH, n (%)	3 (0.3%)	12 (2.0%)	10 (7.0%)	

Abbreviations, BMI, body mass index; FH, familial hypercholesterolemia; HDL-C, high-density lipoprotein cholesterol; LDL-C, low-density lipoprotein cholesterol.

Supplementary Table 4. Participant characteristics based on genetic etiology (n = 1682).

	**No identifiable genetic etiology (n = 1395)**	**Polygenic (n = 262)**	**Monogenic (n = 25)**	***P*-Value**
Age, year	50.38 ± 8.57	49.99 ± 8.30	45.45 ± 9.92	0.015
Male, n (%)	582 (41.7%)	103 (39.3%)	10 (40.0%)	0.761
Non-Whites, n (%)	42 (3.0%)	3 (1.1%)	2 (8.0%)	0.069
BMI ≤ 25 kg/m^2, n (%)	239 (17.1%)	59 (22.5%)	7 (28.0%)	0.170
BMI 25-30 kg/m^2, n (%)	523 (37.5%)	97 (37.0%)	8 (32.0%)	
BMI >30 kg/m^2, n (%)	633 (45.4%)	106 (40.5%)	10 (40.0%)	
Diabetic, n (%)	145 (10.4%)	20 (7.6%)	6 (24.0%)	0.028
Hypertensive, n (%)	515 (36.9%)	79 (30.2%)	10 (40.0%)	0.102
Premature atherosclerotic event, n (%)	245 (17.6%)	55 (21.0%)	7 (28.0%)	0.187
Never smoked, n (%)	819 (59.5%)	168 (64.6%)	13 (52.0%)	0.201
Quit smoking, n (%)	472 (34.3%)	76 (29.2%)	12 (48.0%)	
Current smoking, n (%)	85 (6.2%)	16 (6.2%)	0 (0.0%)	
LDL-C mg/dl	185.72±31.20	191.35±32.18	232.04±54.78	<0.001
LDL-C <190 mg/dl, n (%)	936 (67.1%)	151 (57.6%)	5 (20.0%)	<0.001
LDL-C 190-249 mg/dl, n (%)	410 (29.4%)	99 (37.8%)	12 (48.0%)	
LDL-C ≥250 mg/dl, n (%)	49 (3.5%)	12 (4.6%)	8 (32.0%)	
Triglyceride mg/dl	156.32±67.07	149.40±70.82	133.70±62.38	0.091
HDL-C mg/dl	54.73±14.48	54.63±15.32	58.56±13.55	0.423
Unlikely FH, n (%)	801 (57.4%)	124 (46.6%)	3 (12.0%)	<0.001
Possible FH, n (%)	489 (35.1%)	115 (43.1%)	12 (48.0%)	
Probable/Definite FH, n (%)	105 (7.5%)	27 (10.3%)	10 (40.0%)	

Abbreviations, BMI, body mass index; FH, familial hypercholesterolemia; HDL-C, high-density lipoprotein cholesterol; LDL-C, low-density cholesterol.

Supplementary Table 5. Comparison of mean LDL-C levels in different participant subgroups.

	**Frequency n (%)**	**Mean LDL-C mg/dl**	***P*-value**
Male	695 (41.6%)	185.29 ± 28.03	0.169 A
Female-menarche	240 (14.4%)	188.06 ± 40.60	
Female-menopause	648 (38.8%)	189.20 ± 32.35	
Female-menopause + HRT	87 (5.2%)	187.38 ± 38.38	
Age ≤45 year	401 (23.8%)	185.85 ± 28.65	0.587 A
Age 45 – 55 year	789 (46.9%)	187.60 ± 32.28	
Age >55 year	492 (29.3%)	187.95 ± 35.15	
BMI ≤25 kg/m^2	305 (18.1%)	184.19 ± 30.67	0.102 A
BMI 25-30 kg/m^2	628 (37.3%)	186.95 ± 32.43	
BMI >30 kg/m^2	749 (44.5%)	188.82 ± 32.84	
Whites	1635 (97.2%)	187.28 ± 32.50	0.989 B
Non-Whites	47 (2.8%)	187.35 ± 25.79	
Diabetic	171 (10.2%)	193.38 ± 36.70	0.009 B
Non-diabetic	1511 (89.8%)	186.59 ± 31.73	
Hypertensive	604 (35.9%)	189.07 ± 35.61	0.089 B
Non-hypertensive	1078 (64.1%)	186.28 ± 30.31	
Premature atherosclerotic event	307 (18.3%)	192.70 ± 36.96	0.001 B
No premature atherosclerotic event	1375 (81.7%)	186.07 ± 31.09	
Polygenic score decile 1-9	1391 (82.9%)	185.72 ± 31.20	<0.001 A
Polygenic score decile 10	266 (15.6%)	191.35 ± 32.18	
Monogenic FH	25 (1.5%)	232.04 ± 54.78	

Abbreviations, BMI, body mass index; FH, familial hypercholesterolemia; HRT, hormonal replacement therapy. Test of difference, A, one-way ANOVA test; B, independent t-test.

Supplementary Table 6. Association of demographic and clinical factors with LDL-C level.

	Coefficient (95% CI)		*P*-value
Male	Ref	-	-
Female-menarche	3.36	(-1.651 - 8.377)	0.188
Female-menopause	3.78	(0.142 - 7.414)	0.042
Female-menopause + HRT	2.22	(-5.123 - 9.569)	0.553
Age ≤45 year	Ref	-	-
Age 45 – 55 year	1.67	(-2.476 - 5.814)	0.430
Age >55 year	2.58	(-2.100 - 7.266)	0.279
BMI ≤ 25 kg/m^2	Ref	-	-
BMI 25-30 kg/m^2	3.36	(-1.220 - 7.946)	0.150
BMI >30 kg/m^2	4.43	(-0.076 - 8.944)	0.054
Non-white race	1.03	(-8.400 - 10.467)	0.830
Diabetes	5.84	(0.409 - 11.274)	0.035
Fx of high cholesterol	2.39	(-0.650 - 5.436)	0.123
R-squared	0.011	-	-

Abbreviations, BMI, body mass index; Fx, family history; HRT, hormonal replacement therapy.

Supplementary Table 7. Association of different genetic categories with LDL-C.

	Coefficient (95% CI)		*P*-value
Polygenic score decile 1	Ref	-	-
Polygenic score decile 2	-2.68	(-12.73 - 7.32)	0.601
Polygenic score decile 3	-0.06	(-9.78 - 9.67)	0.991
Polygenic score decile 4	2.05	(-7.22 - 11.31)	0.665
Polygenic score decile 5	3.38	(-5.44 - 12.19)	0.453
Polygenic score decile 6	4.74	(-4.15 - 13.63)	0.296
Polygenic score decile 7	4.90	(-4.17 - 13.96)	0.289
Polygenic score decile 8	7.77	(-0.76 - 16.31)	0.074
Polygenic score decile 9	5.64	(-2.85 - 14.12)	0.193
Polygenic score decile 10	9.57	(1.16 - 17.98)	0.026
Monogenic FH	49.94	(35.28 - 64.60)	<0.001
R-squared	0.044*	-	-

The model is also adjusted for the first 10 principal components of ancestry.
* R-squared rose to 7.4% after incorporation of data regarding all variants into the regression model.
